# Targeted drug delivery systems for pancreatic cancer therapy: advances, challenges, and future perspectives

**DOI:** 10.3389/fimmu.2026.1785625

**Published:** 2026-03-26

**Authors:** Xiaonan Zhang, Xufeng Tao, Yunshu Zhang, Guangshuo Gan, Jing Lv, Lu Zhang, Deshi Dong

**Affiliations:** 1 Department of Pharmacy, First Affiliated Hospital of Dalian Medical University, Dalian, Liaoning, China; 2 Institute (College) of Integrative Medicine, Dalian Medical University, Dalian, Liaoning, China

**Keywords:** clinical translation, immune microenvironment, nanocarriers, pancreatic cancer, targeted drug delivery systems

## Abstract

The review summarizes recent advances, challenges, and future perspectives in targeted drug delivery systems (DDSs) for pancreatic cancer (PC) therapy. Given the dismal prognosis of PC treatment is hindered by a dense desmoplastic stroma, profound hypoxia, limited T-cell infiltration, and abundant immunosuppressive myeloid populations, together forming physical and immunological barriers to effective therapy. Targeted DDSs based on organic, inorganic, and biological platforms (e.g., liposomes/lipid nanoparticles, polymeric nanoparticles, carrier-free drug self-assembly systems, hybrid inorganic–organic nanomaterials, and biomimetic carriers such as exosomes and protein nanocages) can enhance tumor accumulation and reduce off-target toxicity through active ligand–receptor targeting, microenvironment-adaptive delivery, and controlled release triggered by internal cues (pH, enzymes, reactive oxygen species, hypoxia) or external stimuli (light, heat, magnetic fields). Importantly, DDSs are designed to remodel the immunosuppressive tumor microenvironment (TME) by reprogramming tumor-associated macrophages, inhibiting myeloid-derived suppressor cells, activating innate immune sensing pathways, and overcoming stromal immune exclusion via stroma–immune co-modulation or transcytosis-enabled penetration. We further discuss precision-medicine opportunities, proposing biomarker-guided stratification and monitoring frameworks that link patient-specific TME features (e.g., stroma-high/immune-excluded, myeloid-dominant, weak innate priming) to rational DDS selection and combination regimens. Future development should prioritize clinically actionable combination strategies, localized/depot delivery when appropriate, and data-driven design and optimization (including artificial intelligence and machine learning) to accelerate personalized, translatable DDSs for improving pancreatic cancer outcomes.

## 1 Introduction

Pancreatic cancer (PC) remains a highly fatal malignancy with one of the worst prognoses amongst all cancer types (1–4). PC rarely shows early clinical manifestations, and symptoms become apparent when the tumour invades surrounding tissues or metastasizes to distant organs (3, 5). Worldwide, the number of deaths and disability-adjusted life years (DALYs) caused by PC has more than doubled (1). The number of deaths associated with PC is expected to increase by 25% by 2025, making it the third leading cause of cancer-related death in the European Union (EU) after lung cancer and colorectal cancer (6).

**Figure 1:**
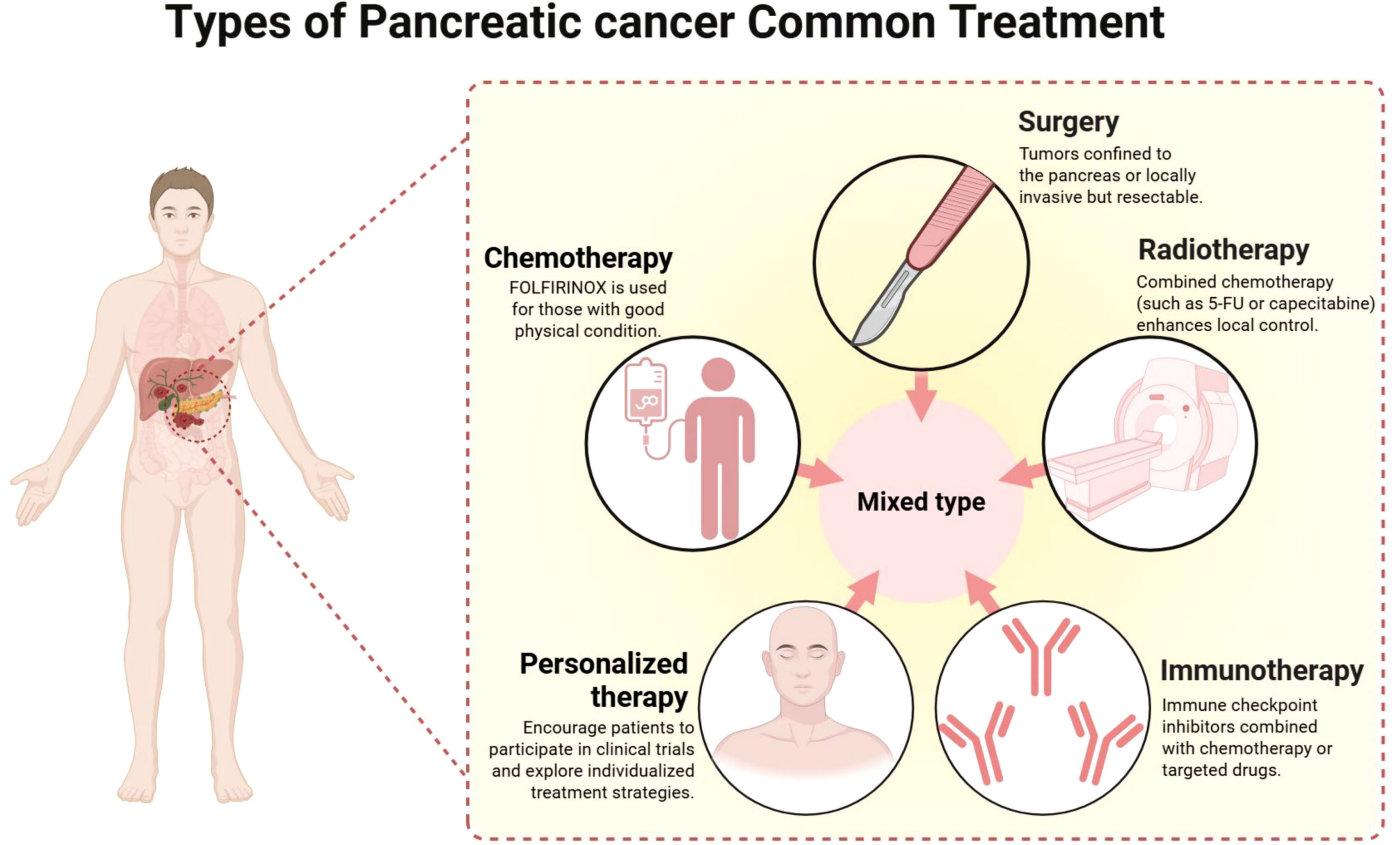
Existing treatment methods for pancreatic cancer. Schematic overview of current PC treatments, including surgical resection, chemotherapy (e.g., FOLFIRINOX), radiotherapy (often with 5–FU/capecitabine), immunotherapy (commonly combined with other modalities), and personalized strategies, these approaches are frequently used in combination depending on disease stage and patient status.

Current treatment methods for PC include surgical resection, chemotherapy, radiotherapy, and immunotherapy, as well as personalized treatment methods tailored to a particular type of PC. However, the efficacies of these treatments remain unsatisfactory (**Figure 1**). The current state of PC chemoresistance, which is caused by the biological complexity and heterogeneity of PC, poor specificity and uneven distribution of chemotherapy drugs, and side effects on normal tissues and organs, limit the effectiveness of conventional chemotherapy regimens, such as FOLFIRINOX and GEM plus nab-paclitaxel. Moreover, difficulties with the clinical application of immunotherapy remain. First, immunotherapy-associated toxicities can arise from on-target/off-tumor recognition (when the targeted antigen is also expressed in normal tissues) and/or non-specific immune activation, leading to immune-related adverse events. Second, the intricate immunosuppressive microenvironment of solid tumours hinders the delivery of immune cells or cytokines to the tumour site through intravenous infusion (7). Thus, although emerging approaches have been investigated to improve PC chemotherapy, few clinical trials have been successful. The development of efficient, safe and targeted strategies has become an urgent challenge for PC treatment.

Drug delivery systems (DDSs) exclusively exploit the vascular abnormalities of tumours to avoid the diffusion of the therapeutic entities into normal tissues and increase the penetration of chemotherapeutic agents into cancerous cells, hence overcoming the drawbacks of conventional therapy (8). Here, we systematically summarize the application of different drug carriers, including nanoparticles, biocarriers, and viral therapies, with the goal of providing more possibilities for PC treatment in the future (9, 10). Prospectively, preclinical and clinical studies should further focus on optimizing drug delivery to develop new therapeutic strategies for PC (**Table 1**) (28).

**Table 1:** Drug carrier specific information.

Specificities	Organic nanoparticles	Inorganic nanoparticles	Biological carriers
Name	Polymer nanoparticles, lipid nanoparticles	Gold Nanoparticles, Ferrite Nanoparticles,	Proteins, Virus
Material composition	Organic polymers or lipids	Metallic or non–metallic materials	Virus, biomolecule: protein or nucleic acid
Preparation Methods	Emulsification, solvent precipitation, etc.	Solvent Precipitation, Reduction synthesis, etc.	Dependent on the biocarrier type (Passed on through biosynthesis or genetic engineering)
Drug–loading Capacity	Moderate drug loading capacity strength	Depends on nanoparticle size and surface properties	Biocarriers can carry with large quantities of drugs
Targeting Properties	Specificity through surface functionalization	Surface Modification to achieve specific target Directionality	Natural targeting
Drug release Controls	pH or temperature response for controlled release	Changing surface properties	Natural Drug Release Mechanisms
*In vivo* behavior	Good biocompatibility compatibility	Surface finishing related	Good biocompatibility compatibility
References	(11–15)	(16–21)	(22–27)

## 2 Organic nanoparticles

### 2.1 Liposomes

**Figure 2:**
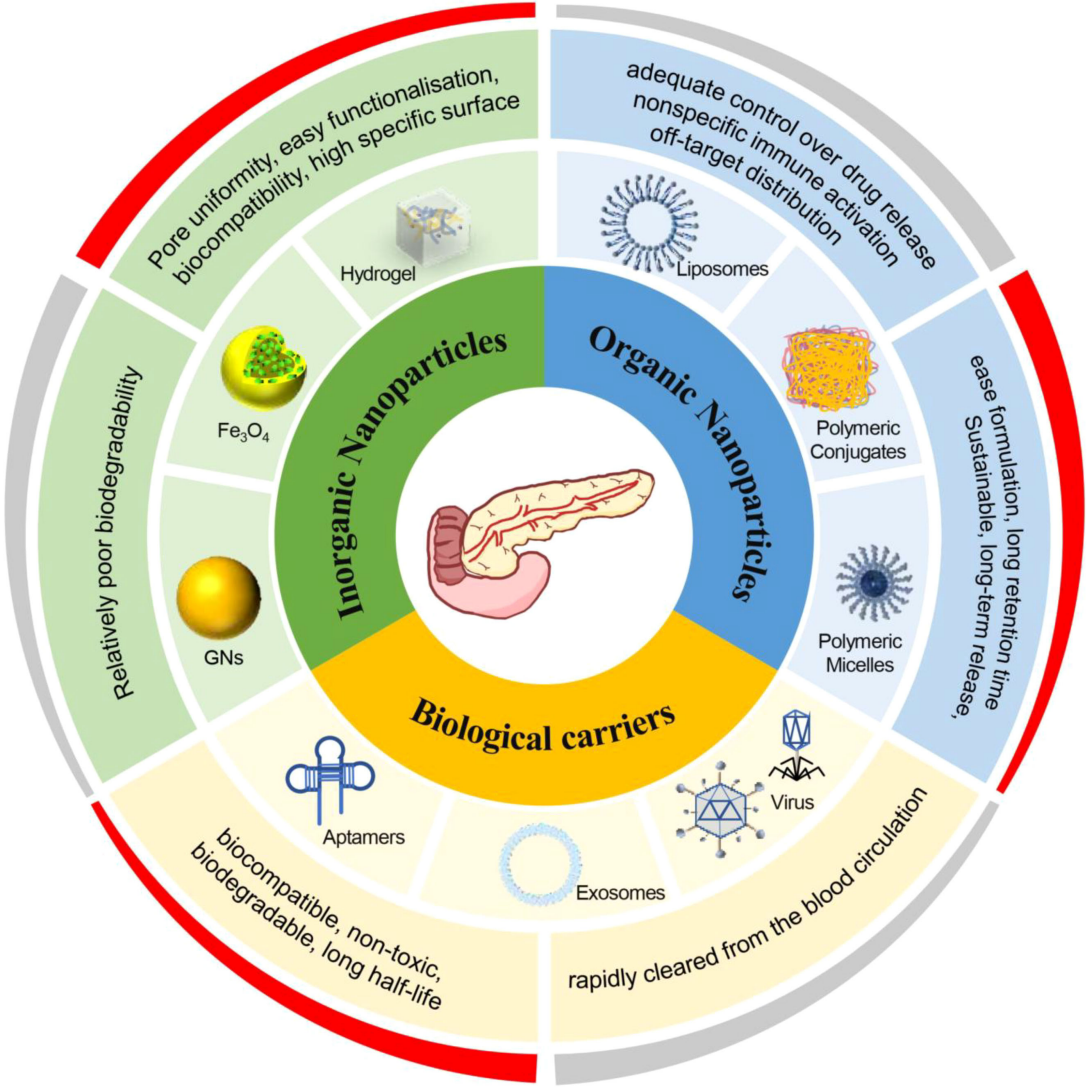
Classification and advantages/disadvantages of targeted drug carriers. Schematic classification of targeted drug delivery carriers for pancreatic cancer into organic nanoparticles (e.g., liposomes, polymeric conjugates, polymeric micelles), inorganic nanoparticles (e.g., gold nanoparticles, Fe_3_O_4_ magnetic nanoparticles, hydrogels), and biological carriers (e.g., aptamers, exosomes, viral vectors). The outer ring summarizes representative strengths and limitations of each class, including controllable/triggered release and formulation simplicity for organic systems, high surface functionalization and structural uniformity but relatively poorer biodegradability for inorganic systems, and high biocompatibility but rapid clearance/scale–up constraints for biological carriers.

**Figure 3:**
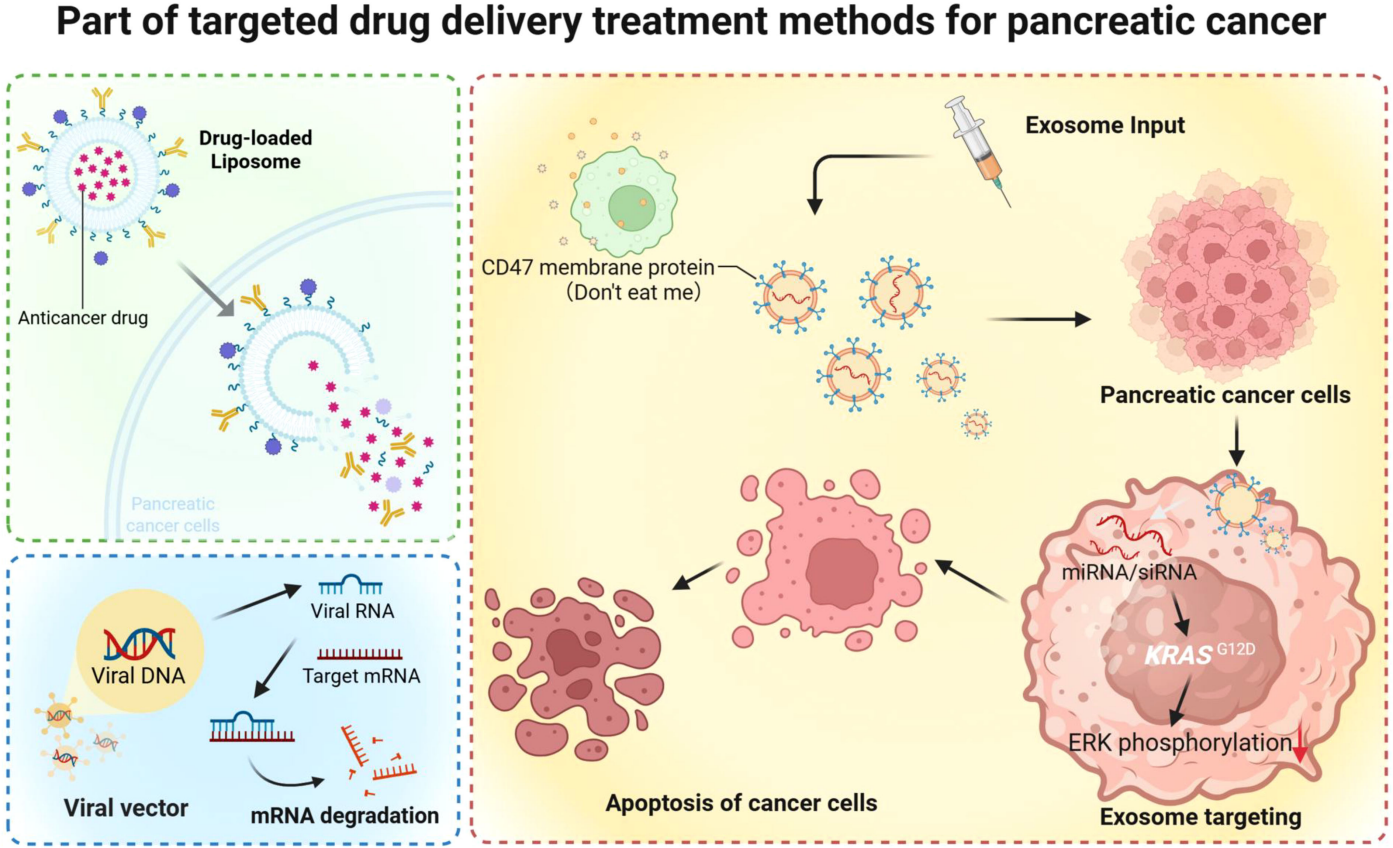
Part of targeted drug delivery treatment methods for pancreatic cancer. Representative targeted DDS strategies for pancreatic cancer, including (left) drug–loaded liposomes delivering chemotherapeutics with controlled release, (bottom–left) viral vectors carrying nucleic acids to silence target mRNA via RNA interference, and (right) exosome–based delivery that exploits CD47 (“don’t eat me”)–mediated immune evasion to enhance circulation and tumor uptake. Exosomes loaded with miRNA/siRNA (e.g., targeting KRAS^G12D^) inhibit downstream signaling (reduced ERK phosphorylation), thereby promoting pancreatic cancer cell apoptosis.

Liposomes are spherical self-assembled nanostructures that range in size from 5–200 nm and are composed of concentric lipid bilayers (29) (**Figure 2**). Depending on the affinity of various vesicle components, lipophilic or hydrophilic drugs encapsulated in either the aqueous phase or the bilayer of liposomes for delivery (**Figure 3**) (30). By adjusting the ratio of the hydrophilic and hydrophobic portions of lipid molecules, the morphology of the vesicle can be altered to increase the loading of different drugs. Depending on the hydrophilicity, hydrophobicity, etc., of the drug, the mode of drug delivery inside the cell varies. Hydrophobic and weakly basic drugs such as adriamycin or vincristine can enter cell as free drugs by passive diffusion in their uncharged form along the concentration gradients, whereas small hydrophilic drugs can be delivered via cell membrane transporter proteins (**Table 2**) (11). Drug release is influenced by serum protein exposure (37). Altering the content of the liposomal bilayer by incorporating cholesterol (12, 38) has been shown to “tighten” the fluid bilayer and reduce the leakage of liposomal contents (11). Liposomes are used as drug carriers to increase the stability and duration of action of drugs and reduce their absorption by normal tissues. Liposomes also increase the solubility of hydrophobic drugs (13, 39). DDSs with unmodified liposomes are limited by their short circulation time, instability *in vivo*, and poor target selectivity (40). Functional modification of liposomes also improves the drug sensitivity at the lesion site and achieves targeted drug release.

**Table 2:** Advances in research on targeted drug carriers.

Drug carriers	Material composition	Type of experiment	Delivery methods	Preparation methods	Drug release control	*In vivo*/*in vitro* behavior	Therapeutic effects	Adverse reactions	Advantages	R
Organic Nanoparticles	Liposomal irinotecan (nal‐IRI)	Clinical Phase III	Intravenous injection	Liposomal encapsulation	NA	NA	The median overall survival of patients treated with nal‐IRI combination with fluorouracil and folinic acid was 6.1 months, significantly higher than that of the other two groups. (6.1 vs. 4.9, 4.2 months)	Neutropenia (27%), Diarrhoea (13%), Vomiting (11%), and fatigue (14%)	Prolonged survival with a manageable safety profile.	(31)
	PEGylated Recombinant Human Hyaluronidase (PEGPH20)	Clinical Phase Ib	Intravenous injection	NA	A standard 3 + 3 dose–escalation design	Prolonged circulating half–life sustains HA enzyme catabolism in tissues and PEGPH20 inhibits tumor growth in a PDA xenograft model.	Median progression–free survival and overall survival were reduced compared to pre–treatment patients with high hyaluronic acid.	Musculoskeletal and extremity pain, peripheral edema and fatigue.	Depletion of intra–tumor hyaluronic acid significantly reduces interstitial fluid pressure and increases drug delivery.	(32)
Inorganic Nanoparticles	Fe_3_O_4_	Cell Experiment	*In vitro* cellular administration	An aqueous dispensable PLGA encapsulated Hsp90 inhibitor (17AAG) and magnetic nanoparticles/nano balls (MNBs) 17AAG to MNPs to polymer as 1:1:10	Differences in polymer concentration affect drug release	Toxicity assays showed a dose–dependent decrease in cell viability of pancreatic cancer cells MIA–PaCa–2 over 48 hours of culture compared to controls.	The 1:1:10 formulation formulations were better in particle characterization with a relative increase in the amount of drug released from the formulation, drug loading.	NO	Differences in polymer concentration, resulting in a relatively increased drug load in the case of the 1:1:10 formulation, exhibited time–dependent sustained cytotoxic effects.	(33)
	Gold nanoparticles (GNs)	Mouse Experiment	Minimally invasive surgical implantation	Biodegradable honeycomb gold nanoparticles composed of liposomes and gold nanoparticles	Interventional Photothermal Therapy (IPTT) combined with Brachytherapy (BT) Interventional Photothermal Therapy (IPTT) combined with Brachytherapy (BT) (IPT–BT)	Accumulation and activation of GNs in deep abdominal tumors as endogenous sensitizers for interventional NIR and internal radiation sources.	High tumor suppression with no significant apoptosis/necrosis of major organs in all groups of mice, by increasing blood flow and oxygen supply, and enhancing the penetration of HGNs at the tumor site.	NO	Local therapy to enhance the energy accumulation of radiation at the tumor site and reduce the damage to normal tissues, *in vivo* HGNs can be excreted from the body through the glomerular basement membrane after treatment.	(34)
Biological Carriers	Adenoviral vectors	Clinical Phase I	Intra–tumor injections	Serotype–containing adenovirus was transfected into 293T cells, screened, propagated, and purified to obtain Ad.IL–12	Ad. IL–12 was administered in doses ranging from 2.5 × 10^10^ to 3 × 10^12^ viral particles	Influences antitumor activity, antiangiogenic effects and immune responses mediated by T cells and natural killer cells.	Dose–limiting toxicity was not reached and no cumulative toxicity was observed.	Exhibited frequent but transient carrier–related adverse reactions, including fever, malaise, sweating, and lymphopenia.	Ease of genetic engineering of small genomes with all gene functions established, high titer production under good manufacturing practices, and specific targeting of dividing and non–dividing epithelial cells, including adenocarcinoma.	(35)
	Retroviral vectors	Clinical Phase I/II	Intravenous injection	Generated by transient co–transfection of plasmids in 293T cells, the final product had a vector titer of 5×10^9^ CFU/ml of Rexin–G vector	Incremental doses of Rexin–G intravenous injection	Surveillance–guided retroviral Rexin–G nanoparticles enter cancerous lesions and increase the concentration of active vectors in the tumor microenvironment through protease activity exposure or tumor invasion.	After one cycle of treatment with Rexin–G, disease stabilization, improved tumor response as well as longer progression–free survival and overall survival were obtained at all dose levels used.	NO	Safe and well tolerated and may improve survival in patients with chemotherapy–resistant metastatic pancreatic cancer.	(36)

Partial abbreviations:

5‐FU/LV: 5‐fluorouracil/leucovorin.

SN–38: 7–ethyl–10–hydroxycamptothecin.

TEAEs: Treatment‐emergent adverse events.

PDA: Pancreatic ductal adenocarcinoma.

17AAG: 17– N–allylamino– 17–demethoxygeldanamycin.

Hsp90: Heat Shock Protein 90.

NIR: Near‐Infrared Region.

HGNs: Honeycomb‐like Gold Nanoparticles.

Lipid-based nanoparticles (NPs) are widely used in biomedical applications because of their simple preparation, biocompatibility, and bioacceptability (41, 42). Liposome encapsulation significantly improves the pharmacokinetic properties of drugs (43–45). After administration of a liposomal gemcitabine, the dose-normalized area under the curve (AUC) in plasma and tumors was 672 times and 1047 times that of free gemcitabine, respectively. The half-life of liposomal gemcitabine was prolonged, and its accumulation in tumor tissues increased significantly (46). In addition, liposomes can be loaded with fragments that improve the PC tumour microenvironment in addition to therapeutic drugs to promote therapeutic effects. For example, heat-sensitive liposomes loaded with doxorubicin (DOX) were modified with low-density membrane type 1 matrix metalloproteinase (MT1-MMP)-activated cilengitide (MC) to produce MC-T-DOX. Cilengitide is released from tumour endothelial cells when MC-T-DOX is activated by MT1-MMP. In addition, MC-T-DOX stimulates angiogenesis in the tumour microenvironment, and the encapsulated DOX becomes more accessible to tumour cells, as only a small stromal distance is needed for DOX to reach tumour cells (47).

Liposomes are known for their compatibility with biological systems and capacity for degradation within the body. Notable strengths of liposomes include their ability to harbour both hydrophilic and lipophilic compounds and effective minimization of the systemic toxicity of the encapsulated medications. Despite these benefits, liposomes face challenges in terms of stability, as liposomes often merge and release drugs before their introduction into circulation. Liposomes also require stringent refrigeration conditions for storage, which can complicate their handling and distribution. However, it is important to note that liposomes nonspecifically adsorb to plasma proteins, making them easily digested and cleared by macrophages, which also results in low targeting efficiency *in vivo* (39).

### 2.2 Polymer nanoparticles

#### 2.2.1 Polymeric conjugates

Polymer delivery systems are defined as formulations or materials capable of introducing therapeutic substances into the body. Polymer nanoparticles derived from natural or synthetic components, which can be either monomeric or preformed polymers, enable the establishment of large formulations to ensure adequate drug delivery. Such materials improve the safety and efficacy of drugs by controlling the rate, location and timing of drug release in the body (48). In contrast to liposomes and nanoparticles, which physically entrap drugs, polymer–drug conjugates can be degraded into smaller fragments in biological systems, ensuring that the carrier is eliminated from the body after drug release (**Table 2**).

A designed oral delivery system was modified with a γ-glutamyl transpeptidase-reactive camptothecin–polymer conjugate to infiltrate the entire tumour. When the conjugate penetrates the luminal endothelial cells of the tumour arteries or extravasates into the tumour stroma, the migration of positive charges along the cell membrane aids in the transendothelial and transcellular transport of the drug and its generally uniform distribution throughout the tumour. In a mouse model of orthotopic pancreatic tumours, the conjugate significantly prolonged survival compared with the first-line chemotherapeutic agent GEM, showing potent antitumour activity (48). In addition, polymeric conjugates not only allow a variety of anticancer drugs to target specific targets but can also be loaded with genes for specific delivery. A natural biodegradable nanostructure can be used for targeted drug delivery and gene therapy and in pharmaceutical, biomedicine, and nanoarchitecture applications with high transfection efficiency and low cytotoxicity (14).

The design of polymer–drug conjugates stands out owing to the direct linkage of the therapeutic agents to polymers, which increases the therapeutic index and fosters controlled drug release. However, the intricacies of the chemical structures of such conjugates can be a double-edged sword and potentially complicate the synthetic process and lead to unpredictable drug release patterns.

#### 2.2.2 Polymeric micelles

As self-aggregating colloidal systems, polymeric micelles are a type of nanocarrier prepared from amphiphilic polymers with an internal lipophilic core and an external hydrophilic shell. Compared with other nanocarriers, micelles are smaller and enable passive targeting (even of poorly permeable tumours) (49), improve cellular internalization (50), and allow suitable assimilation of hydrophobic compounds. The naturally hydrophilic nature of polymeric micelles extends the blood circulation time (49, 51). Particularly attractive features of micelles include their ease of preparation and their great scaling potential compared with other nanocarriers, such as polymeric nanoparticles and liposomes, which require more complex, longer, and expensive manufacturing procedures (15, 50, 52).

An attractive strategy for improving the *in vivo* stability of GEM and its delivery to tumours is bioconjugation to a polymeric carrier. Studies have demonstrated a significant increase in the bioavailability of GEM when it is conjugated with polyethylene glycol (PEG), lipids, and squalene derivatives (53–55). Polycarbonates are biodegradable and exhibit low toxicity, as their degradation products include carbon dioxide and alcohols, which have a lesser effect on the pH of the microenvironment and do not cause local inflammation (56). Researchers have successfully resolved the problems of poor solubility and rapid uptake by the reticuloendothelial system (RES) while decreasing the GEM payload by synthesizing a copolymer with a biocompatible PEG block and a biodegradable PCC block (57). Xenograft tumours of MIA PaCa-2 cells (human PC cells) were inhibited after systemic administration of this copolymer by modifying micelles with numerous carboxyl pendant groups. In an orthotopic mouse model of pancreatic tumour progression, mixed micelles containing peptides linked to the GE11 peptide efficiently delivered GEM to PC cells expressing epidermal growth factor receptors (EGFRs) and interacted with tumour blood vessels to inhibit pancreatic tumour growth (58).

Additionally, polymeric micelles have been fabricated to deliver small-molecule chemotherapeutic agents such as oligonucleotides and small-molecule antibiotics in place of conventional drugs such as GEM (59). Studies have proven that miRNAs and hydrophobic drugs loaded within polymer micelles exhibit antitumour effects and overcome the disadvantages of off-target effects, low transfection efficiency, poor water solubility and burst release when drugs are used alone (60). Good biodistribution and anticancer efficacy were reported by Kumar et al. in excellent detail. Their micelles improved the biodistribution of polymeric micelles coformulated with the polyanionic hydrophilic miRNA let-7b and the small-molecule hydrophobic drug GDC-0449, leading to decreased renal clearance and increased circulation time in plasma (61). Micelles loaded with volasertib and coated with drug particles by N–B coordination with a positively charged surface formed a complex with miR-34a via electrostatic interactions. The hydrophilic character of the micelles extended the circulation of volasertib and miR-34a, resulting in increased accumulation in the tumour area (60). However, polymeric micelles have certain limitations, as they are not always biocompatible, and complex biosynthesis can impede production.

### 2.3 Drug self-assembly nanoparticles

Drug self-assembly nanoparticles are constructed through various self-assembly mechanisms. Drug molecules can be chemically modified to enable spontaneous nanoassembly through noncovalent interactions (π-π stacking, hydrogen bonding, hydrophobic interactions), metal ion coordination, or combinations of covalent bonds and noncovalent forces (62). For pancreatic cancer, by successfully utilizing PUFAylation technology, gemcitabine is combined with hydrophobic linoleic acid to self-assemble into nanoparticles smaller than 100 nanometers. The treatment results are superior to those of free gemcitabine, significantly inhibiting tumor progression and reducing systemic toxicity (63). In addition, a carrier-free nanoparticle based on the self-assembly of curcumin-erlotinib conjugate (EPC) exhibits stronger cytotoxicity, better anti-migratory and anti-invasive effects on BxPC-3 pancreatic cancer cells. Moreover, in a mouse model of pancreatic tumors, the growth of pancreatic tumors is inhibited and no systemic toxicity is detected (64). The uniformity of carrier-free drug conjugates and ease of scaling up production offer advantages for clinical translation compared to complex multi-component nanocarriers (65).

## 3 Inorganic nanoparticles

Inorganic nanoparticles are usually metal-based, such as gold nanoparticles and iron oxide nanoparticles. Inorganic nanoparticles are characterized by various physical, electrical, magnetic, and optical properties. Emodin (EMO) is a natural product widely used for tumour therapy. Magnetic nanoparticles (MNPs) packaged in a PEG-modified phospholipid micelle structure coupled with emodin and stable iron oxide nanoparticles (magnetite, Fe_3_O_4_) (**Table 2**) can improve the hydrophilicity and surface tension of PEG. Fe_3_O_4_-PEG-Cy7-EMO NPs have many advantageous properties for cancer treatment, including increased magnetic susceptibility, improved biocompatibility, passive targeting of PC cells, improved drug loading and release behaviour, and improved efficacy against tumours (16). In addition, magnetic nanoparticles can effectively deliver an MRI contrast agent to the target, which helps in the diagnosis of PC (17).

In the clinic, paclitaxel (PTX)/GEM nonmetallic inorganic mesoporous silica nanoparticles (MSNPs) have been shown to bypass matrix barriers, improve GEM pharmacokinetics, and allow for the simultaneous delivery of synergistic drug combinations to advance PC nanotherapies (18–20, 66–71). Researchers have coloaded PTX and GEM into MSNPs coated with lipid membranes, and the chemical makeup and hydrophobicity of the drugs, the efficacies of their combinations at the best dosage ratios, and the drug-loading capacity and rapid, high encapsulation efficiency have been studied. Compared with GEM-loaded LB-MSNPs and free GEM, intravenously injecting PTX/GEM-loaded LB-MSNPs into the veins of mice bearing subcutaneous PANC-1 xenografts resulted in more significant tumour shrinkage, effective inhibition of primary tumour growth and the elimination of metastatic foci (72).

Semiconductor quantum dots are nanoscale light-emitting particles with unique photophysical properties. Quantum dots have been applied in the field of cell biology as drug vehicles for synthetic nanoprobes with fluorescent imaging functions and become physically connected to the surface of cancer cells to inhibit cell growth as integrin receptor antagonists. Encouragingly, a recent study reported a relationship between PEG–PEG chain length and quantum dot accumulation in the pancreas, which may lay a foundation for the development of more specific targeting agents that are particularly important for pancreatic tissues with few molecular targets (21). Joshi et al. designed a better alternative to semiconductor graphene quantum dots and evaluated their efficacy in PC bioimaging and drug delivery. These quantum dots have excellent fluorescence performance and are cost effective, and the biocompatibility of their nanoscale formulations can ensure specific drug delivery to tumour sites after loading high contents of drugs (73).

Inorganic nanoparticles have distinct electronic and optical characteristics, making them particularly suitable for diagnostic imaging and therapeutic interventions. They also typically demonstrate superior stability. However, the lack of extensive data on the long-term biocompatibility and safety of these materials is a prominent concern. Their propensity for accumulation in the body raises additional questions regarding their long-term impact on human health.

Hybrid inorganic–organic nanoparticles integrate the distinctive physicochemical advantages of inorganic cores (e.g., magnetic, optical, porous, or high-density materials) with the biological functionalities of organic components (e.g., lipids, polymers, peptides, or biomembranes) (74, 75). A paradigm-shifting approach involves inorganic-organic hybrid nanoparticles with drug molecules as structural anions, achieving extraordinarily high drug loading. Ischyropoulou et al. developed [ZrO]²^+^[GMP]²^−^ IOH-NPs containing gemcitabine monophosphate (GMP) as the drug anion, achieving 76% drug load by mass—far exceeding conventional nanocarriers (74). GMP-IOH-NPs have advantages such as high drug loading capacity, tumor-specific delivery, enhanced therapeutic efficacy, and prevention of gemcitabine inactivation. Meng et al. demonstrated that lipid-coated MSNs co-delivering gemcitabine (40 wt% loading) and paclitaxel achieved synergistic tumor shrinkage in PANC-1 xenografts, with comparable efficacy requiring 12-fold less drug than free Abraxane plus gemcitabine (72). Milk protein (casein)-coated magnetic iron oxide nanoparticles (CNIO) conjugated with urokinase plasminogen activator amino-terminal fragment (ATF) and cisplatin achieve ~25 wt% drug loading with sustained release (76). ATF-CNIO-CDDP can serve as an effective integrated diagnosis and treatment platform for actively targeted enhancement and image-guided cancer therapy, while reducing systemic toxicity. The organic shell of inorganic-organic hybrid nanoparticles (such as lipid or polymer coatings) enhances system stability and cycling capability, while the inorganic core (like mesoporous silica or metal-organic frameworks) enables high drug loading and efficient co-delivery. In addition, hybrid materials can utilize their own magnetic or optical inorganic components to promote theranostic and stimulus-responsive capabilities, further achieving controlled release or on-demand release.

## 4 Biological nanoparticles

In recent years, interest in biomedicine has increased because of the renewability, nontoxicity, biocompatibility, biodegradability, lengthy blood circulation duration, and targeting ability of natural biological carriers. The biological functions of biological carriers of natural origin have become better understood due to extensive studies. Such research suggests that biological drug carriers may have some advantages over synthetic material-based carriers in terms of their half-life, stability, safety, and ease of manufacture (**Figure 2**) (77).

### 4.1 Aptamers

An aptamer is a single-stranded oligonucleotide that can bind tightly and selectively to target molecules by folding into specific structures. A variety of molecules, including enzymes, antibodies, cell surface proteins, bacteria, parasites, viruses and mammalian cells, have been selected as targets for aptamers (22–24). The XQ-2d aptamer can specifically identify and bind the target molecule transferrin receptor 1 (TfR1 or CD71) on the surface of PC cells. After conjugation with the cell-penetrating fusion protein (Arg) 9-SH2 (a broad-spectrum inhibitor of the phosphotyrosine (pY) signalling pathway), the resulting XQ-2d aptamer-based complex can accurately target pancreatic stellate cells (PSCs) and PC cells; eradicate the dense PC matrix; promote the delivery of conjugates to tumour cells; and inhibit the proliferation, metastasis and invasion of PDAC cells. Precise guidance by the aptamer prevents the drug from entering normal tissues and cells, thereby minimizing side effects and contributing to high drug concentrations around the tumour tissues (78). As an activator of a strong inhibitor of cell proliferation (p21), the epigenetically silenced transcriptional factor C/EBPα (CCAAT/enhancer-binding protein α) is upregulated by small activating RNA (saRNA) in PC cells. PC-specific 2′-F-RNAs, which colocalize with C/EBPα-saRNA via a sticky bridge sequence, serve as a targeting modality to deliver C/EBPα-saRNA into PC cells to induce C/EBPα expression. The tumour burden was significantly reduced when aptamer–saRNA conjugates were used in xenograft models.

Aptamers are known for to have high affinity and specificity for their targets, allowing precise targeting of specific proteins or cells. These properties have contributed to the development of highly selective therapeutic strategies. However, the clinical use of aptamers is limited by their susceptibility to degradation by nucleases and possible renal clearance, both of which shorten their therapeutic window.

### 4.2 Exosomes

Exosomes are composed mainly of proteins, lipids and nucleic acids and are surrounded by a lipid bilayer. Exosomes are the smallest type of extracellular vesicles, have a small spherical structure of approximately 50–150 nm and have been isolated from certain patient cell types or multiple body fluids, such as plasma, serum, urine, breast milk, and saliva (25, 26). Exosomes are transferred between cells by molecular interactions for signal transmission and substance delivery and interact with targeted peptides, antibodies and other biomolecules for PC diagnosis and treatment (25). Exosomes have many advantages, such as no cytotoxicity, few side effects, low immunogenicity, natural biocompatibility, high stability during drug delivery, and high cellular uptake (25, 79, 80). Exosome-targeted tissues have strong intrinsic activity, a unique structure, and suitable physicochemical properties (81). These properties ensure the success of local and cell–cell communication to remodel the tumour microenvironment and mediate tumour angiogenesis, differentiation, apoptosis and metastasis.

Endogenous exosomes are considered “ideal” DDSs because they can be combined with other nanoparticles to form stable, safe and effective complexes with a longer half-life and greater efficiency (82). Unlike synthetic nanoparticles, exosomes are nonimmunogenic and noncytotoxic when purified from compatible cell sources. In contrast to liposomes, exosomes include a range of transmembrane and membrane-anchored proteins, which increases their half-life in blood circulation by avoiding phagocytosis, allowing for improved cellular absorption and subsequent delivery of their contents (83). The enhanced retention of exosomes in circulation is due to protection from monocytes and macrophage phagocytosis mediated by the surface membrane protein CD47. A study designed exosomes, denoted iExosomes, from normal fibroblast-like mesenchymal stromal cells to carry siRNAs or shRNAs specific for oncogenic KrasG12D, a common mutation in PC. iExosomes targeting oncogenic Kras are dependent on CD47, and CD47-SIRPα binding initiates “don’t eat me” (84) signal to inhibit phagocytosis facilitated by macrophages, significantly reducing KrasG12D mRNA levels and phosphorylated extracellular regulated protein kinase (ERK) protein levels in Panc-1 cells and showing greater efficacy than liposomes (**Figure 3**). Moreover, the membrane-anchored proteins and plasma membrane-like phospholipids of exosomes may help to prevent their clearance from circulation (85–88). Exosomes containing CRISPR/Cas9 plasmid DNA are transferred to recipient cancer cells to induce deletion of the mutant Kras gene within PC cells by the specific transfer of nonautologous exosomes encapsulating these plasmids (83). A living cell can release exosomes naturally when anticancer drugs and other molecules are delivered to the target PC cells in the endocytic compartment (89). Exosomes derived from PC cells inhibit PC progression by activating the immune system. PC cells or hepatic stellate cells can release exosomes that play a role in both PC pathogenesis and the tumour microenvironment, facilitating the establishment of a suitable microenvironment for PC (90). In addition, a team designed “Smart Exosomes”, which display both RGD and CD47p110–130, through CD9 engineering to increase the binding of αvβ3 to PDAC cells, blocking interactions between the ECM and cancer cells and disrupting protumour signalling pathways (91). Recent research reveals the dual applicability of exosomes in cancer therapy, but the underlying molecular mechanisms remain largely unknown.

As natural nanoscale vesicles, exosomes facilitate intercellular communication and have an intrinsic ability to transfer proteins, lipids, and nucleic acids. Their endogenous origin endows them with excellent biocompatibility and a reduced likelihood of eliciting immunogenic responses. Nonetheless, the technological challenge of isolating and characterizing exosomes with high purity and yield — as well as understanding their complex cargo-loading mechanisms — pose considerable barriers to their widespread therapeutic application.

### 4.3 Virus-mediated gene delivery

A significant body of research has demonstrated that PC initiation and progression can occur through the activation of oncogenes and the inactivation of tumour suppressor genes (92). Viral vectors, one of the main approaches used to efficiently deliver genes for the release of genetic material into target cells, have been applied to target PC cells, including adenoviruses, retroviruses, adeno-associated viruses, reoviruses, and herpes simplex virus (93) (**Figure 2**). There are several targets for PC gene therapy, including the tumour suppressor gene p53, the mutant oncogene Kras, and the antiangiogenic gene VEGFR, among others (94). Without the need for physical or chemical intervention, the virus can enter cells to transfer DNA, and the therapeutic gene can enter the nucleus for integration into the host gene pool (**Figure 3**) (94–96).

#### 4.3.1 Adenovirus

An adenovirus (Ad) is a nonenveloped virus with a 36 kb genome of double-stranded DNA and a capsid that makes it distinct from other viruses (97). An Ad vector has a transgene capacity of approximately 35 kilobases (kb) with high transfection efficiency (**Table 2**). Ad vectors can be purified in high titres to infect both dividing and nondividing cells (92). Over 51 serotypes with distinct tissue tropisms have been identified to date, but the Ad5 vector (serotype 5) is most frequently used because of its ability to infect a wide range of cells (92). A comparison of the transfection efficiency of a conventional type 5 Ad vector (Ad5GFP) with that of chimeric type 5 and 35 fibre proteins (Ad5/35GFP) revealed the greater transfection efficiency of the Ad5/35GFP vector (27). In particular, PC cells express low levels of coxsackie adenovirus receptor (CAR), which may result in low Ad entry (98, 99).

#### 4.3.2 Adeno-associated virus

Adeno-associated viruses (AAVs), which are composed of a protein shell that surrounds and protects a small, single-stranded DNA genome of approximately 4.8 kb, can deliver DNA to target cells (100). It is possible to deliver *in vivo* pancreatic adenoviruses or adeno-associated viruses (rAAVs) using recombinant adenoviruses that are predominantly episomal and designed to deliver large amounts of material. AAV vectors can target specific cell types without altering pancreatic functions or producing humoral responses. Neither normal mice nor PC mice developed pancreatic intraepithelial neoplasia/fibrosis when AAV was injected intraductally, indicating that AAV efficiently and safely targets the pancreas (101).

When used as gene carriers, viruses can efficiently deliver genetic materials and target specific cells with remarkable intracellular transport abilities. Nevertheless, their application is hindered by the potential for immunogenic reactions, oncogenic risks, and the potential for genetic alterations or recombination, which necessitates their meticulous investigation prior to clinical use.

#### 4.3.3 Other virus-mediated biological carriers

Retroviruses are enveloped viruses that contain a positive-sense RNA genome of approximately 7–12 kb (**Table 2**) (92, 93, 102). Gene delivery to PC cells has been efficiently achieved by retroviral vectors (93). An ancient endogenous retrovirus, human endogenous retrovirus-K, has been integrated into the human genome to prevent PC cell proliferation, as well as tumour growth and metastasis in xenograft models (103). In addition, the photodynamic effects of reovirus combined with protoporphyrin IX (PpIX) in the treatment of various human PC cell lines are still under investigation (104). In PDAC cells, pelareorep, an intravenously delivered oncolytic reovirus, induces a T-cell-inflammatory phenotype after administration. During treatment in a phase 1b single-arm trial, new T-cell clones were detected by genome sequencing of peripheral blood T-cell receptors (105). As a herpes simplex virus, VG161 has multiple synergistic antitumour immunomodulatory properties. The antitumour potential of VG161 has been demonstrated through the systematic activation of acquired and innate immunity as well as improvements in the tumour immune microenvironment in PC models (106). The advantages of albumin-based DDSs exploit the advantages of this natural serum protein, including a long half-life in circulation and the ability to bind various substances. Albumin-based DDSs can carry drugs throughout the bloodstream and are well suited for the passive targeting of tumours through the enhanced permeability and retention (EPR) effect. Nevertheless, the integration of drugs into the albumin structure can be complex, and certain drugs may dissociate prematurely or fail to reach their intended targets owing to the intricacies of protein dynamics.

### 4.4 Other biological carriers

Proteins act as natural, biological macromolecule carriers in organisms to deliver drugs with the aim of treating PC (107–109). The natural protein carrier albumin, which circulates in the blood for a long time, can prolong the circulation half-life of drugs that are cleared rapidly and, more importantly, promote their accumulation within tumours (110). Additionally, albumin interacts with receptors that are overexpressed in diseased tissues and cells, providing a unique feature that enhances the ability to target specific disease sites without the need to add specialized ligands (111). In a phase I/II clinical trial with a 28-day dosing cycle, Gemcitabine plus nanoparticle albumin-bound (NAB) paclitaxel (GA) significantly improved six-month survival in patients with metastatic PDAC and a Karnofsky performance status (PS) of at least 70%. Patients with metastatic PDAC and a poor PS also benefitted from the combination of NAB-paclitaxel and GEM, achieving acceptable safety and efficacy outcomes (112).

As a natural polysaccharide, hyaluronic acid (HA) has excellent biodegradability, biocompatibility and nonimmunogenicity (113). HA is a nonsulfated glycosaminoglycan (GAG) present in the extracellular matrix (ECM) of many soft connective tissues (114). Several studies have proven that HA can target specific cells by binding with cell surface receptors such as CD44 and the receptor for HA-mediated motility, making HA a very promising tumour drug delivery agent (115–117). Nanomicelles engineered with HA and loaded with 3,4-difluorobenzylidene curcumin have been explored to kill CD44^+^ stem-like PC cells (118). This was the first example of a natural polymeric drug carrier to successfully deliver a hydrophobic cancer drug into cancer cells, inhibiting the proliferation of and colony formation in PC cells (119). This naturally occurring biopolymer is characterized by its biocompatibility and biodegradability, making it an appealing vehicle for drug delivery applications. It is particularly adept at targeting CD44 receptors, which are overexpressed in various tumour cells, thereby allowing for site-specific drug delivery. However, the quick turnover of hyaluronic acid in the body is a drawback, and chemical modifications may be needed to increase the stability of HA and ensure sustained drug release.

Ferritin nanocages are spherical protein assemblies composed of 24 subunits that self-assemble into a hollow structure with an outer diameter of 12 nm and an interior cavity of 8 nm, providing a natural compartment for drug encapsulation (120). This receptor-mediated targeting enables selective delivery of cargo molecules to tumors followed by rapid internalization. Multiple studies demonstrate the therapeutic potential of ferritin nanocages in pancreatic cancer models. A ferritin-based delivery system for KRAS G12D inhibitor MRTX1133 achieved superior efficacy over free drug in both 2D cell culture and 3D spheroid models, with enhanced cell death and KRAS pathway inhibition in patient-derived organoids (121). Enzyme-responsive biomimetic ferritin nanoparticles (MMFn) engineered with MMP-2/9-responsive peptides demonstrated robust antitumor activity in Panc02 pancreatic cancer xenograft models without systemic toxicity (122). The unique nanocage structure combined with intrinsic tumor-targeting properties positions ferritin-based formulations as promising next-generation nanomedicines for pancreatic cancer therapy.

In addition, engineered bacteria have emerged as “living” delivery vectors leveraging tumor tropism and preferential colonization in hypoxic regions. For instance, the probiotic E. coli Nissle 1917 was engineered/used to deliver p53 and Tum-5 to tumor hypoxic areas (123). Engineered bacteria can not only inhibit the growth of human liver cancer cells but also do not cause obvious side effects in mice, laying a foundation for targeted tumor therapy.

## 5 Nanotechnology-enabled immunotherapy: reprogramming the tumor microenvironment

PC presents one of the most formidable immunosuppressive tumor microenvironments (TME) in oncology, characterized by dense desmoplastic stroma, profound hypoxia, limited CD8^+^ T cell infiltration, and abundant immunosuppressive myeloid populations including tumor-associated macrophages (TAMs) and myeloid-derived suppressor cells (MDSCs). This unique TME architecture creates a physical and immunological barrier that renders PC largely refractory to conventional immunotherapies that have succeeded in other malignancies (124–128).

The failure of conventional immunotherapy in PC stems from three interconnected barriers: cellular immunosuppression mediated by TAMs, MDSCs, and CAFs, physical stromal barriers preventing drug and immune cell penetration, and metabolic reprogramming creating a hostile microenvironment. Nanotechnology-based drug delivery systems (DDSs) offer unique advantages to address these barriers through: TME-responsive drug release triggered by pH, hypoxia, or enzymes, active targeting of specific cell populations, and co-delivery of multiple therapeutic agents for synergistic effects (124, 129–133).

### 5.1 Reprogramming TAMs

TAMs represent the most abundant immune cell population in PC and exist predominantly in an immunosuppressive M2 phenotype (134, 135). Lipid nanoparticles (LNPs) delivering IRF5 mRNA can reprogram M2 macrophages to the anti-tumor M1 phenotype, characterized by increased IL-12 and TNF-α secretion and enhanced antigen presentation. Injectable thermosensitive hydrogels loaded with IRF5 mRNA/CCL5 siRNA nanoparticle complexes achieve sustained local delivery, resulting in significant M1 macrophage enrichment and subsequent T cell-mediated immune responses (136). Secondly, nanoparticle-based delivery of CD40 agonists activates macrophages to promote antigen presentation and cytotoxic T cell priming. This approach has shown clinical promise when combined with chemotherapy (136). In addition, blocking the CCL2/CCR2 axis prevents monocyte recruitment and M2 macrophage accumulation. Nanoparticles can deliver CCR2 inhibitors or anti-CCL2 siRNA to reduce TAM infiltration (137, 138).

### 5.2 Targeting MDSCs

Simultaneously, as the most potent immunosuppressive cell populations in PC, MDSCs are exerting their effects through three primary mechanisms: restriction of dendritic cell maturation and antigen presentation, production of immunosuppressive enzymes and reactive species (ARG1, ROS, NO), and direct inhibition of T cell function via metabolic depletion and oxidative stress, thereby preventing effective T cell priming (136, 139, 140).

Arginase-1 is a key driver of immune suppression in PC. Genetic deletion of Arg1 in macrophages delays invasive disease formation and increases CD8^+^ T cell infiltration, while pharmacological arginase inhibition (CB-1158) further enhances CD8^+^ T cell infiltration and sensitizes tumors to anti-PD-1 checkpoint blockade. MDSCs also produce reactive oxygen species (ROS) through NADPH oxidase, which directly impairs T cell function and contributes to the oxidative stress characteristic of the PC TME (141–143).

Beyond T cell suppression, MDSCs actively inhibit dendritic cell (DC) maturation and antigen presentation through multiple mechanisms. PMN-MDSCs block DC cross-presentation of tumor antigens without affecting direct antigen presentation, an effect mediated by myeloperoxidase (MPO)-driven lipid peroxidation and transfer of oxidized lipids to DCs (144). MDSCs also sequester cystine via the xc- transporter but lack the ASC transporter needed to export cysteine, thereby depriving T cells of this essential amino acid required for activation (145). This metabolic competition with antigen-presenting cells further impairs T cell priming and effector function.

#### 5.2.1 Blocking chemotactic axes: disrupting MDSC recruitment

MDSCs are recruited through GM-CSF, CXCL12, and other tumor-derived chemokines (136, 146, 147). Nanoparticles delivering CXCR4 inhibitors or anti-CXCL12 siRNA disrupt MDSC recruitment. Combined delivery of calcipotriol and anti-CXCL12 siRNA in polyamino acid nanoparticles reduces both ECM deposition and MDSC infiltration, converting “cold” tumors to “hot” tumors responsive to checkpoint blockade (148). Some studies indicate that nanoparticles targeting lactate production (via glycolysis inhibitors or KRAS inhibitors) reduce protein lactylation, which drives MDSC recruitment through CCL2 secretion. Inhibiting lactate-induced ENSA-K63 lactylation disrupts STAT3/CCL2 signaling, reducing MDSC accumulation and enhancing immune checkpoint blockade efficacy (137).

#### 5.2.2 Functional inhibition: suppressing MDSC immunosuppressive mechanisms

Nanoparticles can deliver agents that directly inhibit the enzymatic and metabolic pathways responsible for MDSC immunosuppression, particularly targeting ARG1, ROS, and NO production (139). Gold nanoparticles (Au NPs) modified with MDSC-targeting peptides (H6) disrupt NLRP3 inflammasome assembly in MDSCs by scavenging ROS and abrogating NLRP3-NEK7 interactions. This inhibits IL-1β production—a key cytokine that dampens antitumor immune responses and promotes MDSC-mediated immunosuppression. The 30 nm Au NPs selectively target MDSCs in the TME, reducing both IL-1β levels and MDSC populations while enhancing T cell activation and improving efficacy of PD-1 checkpoint blockade in both sensitive and resistant tumor models (149). Lipoprotein-mimetic nanoparticles targeting scavenger receptor type B-1 (SCARB1), which is specifically expressed on MDSCs, directly inhibit MDSC suppressive activity (150).

### 5.3 Activating the cGAS-STING pathway

The cGAS-STING pathway is a critical innate immune sensing mechanism that can bridge innate and adaptive immunity in PC. STING pathway activation represents a critical strategy to transform immunologically “cold” PC tumors into “hot. STING activation triggers phosphorylation of TBK1 and IRF3, along with NF-κB activation, culminating in robust production of type I interferons (IFN-α/β) and proinflammatory cytokines (IL-6, TNF-α, CXCL10) (151–154). IFN-I signaling enforces tumor antigen presentation on dendritic cells and macrophages, driving their maturation into professional antigen-presenting cells capable of cross-priming CD8^+^ T cells (154, 155). Critically, PC tumors typically exhibit primary resistance to anti-PD-1/PD-L1 therapies due to the absence of pre-existing T cell immunity (134, 156). STING agonists address this limitation by generating tumor-specific T cells that can then be unleashed by checkpoint inhibitors. In preclinical PDAC models, combining STING agonists with anti-PD-1 or anti-PD-L1 antibodies produces synergistic antitumor effects, with response rates and survival significantly exceeding either monotherapy (153, 157–160). This synergy extends to human PDAC samples, where STING and TLR4-mediated type I interferon signaling correlates with enhanced natural killer and CD8^+^ T cell immunity (153).

Nanoencapsulation dramatically improves the circulation half-life of STING agonists. Polymersome nanoparticles (STING-NPs) increase the half-life of encapsulated cGAMP by 40-fold, allowing sufficient time for tumor accumulation via the enhanced permeability and retention (EPR) effect (161, 162). Emerging innovations include stimuli-responsive delivery systems that release STING agonists in response to TME characteristics (acidic pH, hypoxia, ROS, specific enzymes), enabling precise spatiotemporal control of immune activation (163, 164). By designing pH-responsive polymer nanocarriers to promote cytoplasmic delivery (165). Meanwhile, it produces a synergistic effect with immunogenic cell death (ICD). Hydrogel-based *in situ* vaccines co-delivering cGAMP nanoparticles with ICD inducers demonstrate that ICD-derived signals synergistically enhance STING pathway activation, fostering robust DC and CD8^+^ T cell responses that suppress tumor progression across immunologically cold tumor models (154, 166).

### 5.4 The stromal barrier: a multidimensional immune exclusion mechanism

The PC TME comprises up to 90% non-neoplastic cells, with cancer-associated fibroblasts (CAFs) producing extensive extracellular matrix (ECM) rich in collagen, hyaluronic acid, and fibronectin. The stroma functions as a multifaceted “immune barrier” that simultaneously prevents drug delivery and excludes cytotoxic T cells from reaching tumor cells, creating an immunologically “cold” microenvironment that drives therapeutic resistance (134, 135, 167–169). The PC stroma functions as a physical barrier through multiple mechanisms that actively prevent T cell-tumor contact. Dense collagen networks create a physical impediment that restricts T cell migration (170). Stromal fibrin further shapes the immune infiltration landscape by acting as both a physical barrier and biochemical niche that restricts CD8^+^ T cell and tumor-associated macrophage penetration from the tumor stroma into the tumor parenchyma (171). Hypoxia induces HIF-1α, which inhibits T cells through the PD-1/PD-L1 axis, while the physical parameters of the ECM—including stiffness, pressure, and mechanical stress signaling—actively shape the immunosuppressive microenvironment (134).

Hyaluronidase (HAase)-loaded nanoparticles directly degrade hyaluronic acid, a major ECM component that contributes to elevated interstitial pressure and impaired drug delivery. Anti-PD-1-conjugated ZIF-8 nanoparticles co-loaded with HAase and decitabine (DEC) demonstrate a self-reinforcing infiltration loop: HAase degrades stroma while DEC induces CCL5 secretion, which recruits additional nanodrug-loaded tumor-infiltrating lymphocytes (TILs) for further HAase and DEC release. This approach increased TIL infiltration by 12-fold in immunodeficient mice and enabled tumor eradication with 10-fold lower TIL doses than conventional therapies (172). Transcytosis-based strategies enable nanoparticles to cross the stromal barrier while preserving stromal integrity. Enamine N-oxide-modified nanoparticles co-loaded with gemcitabine prodrug and galunisertib (TGF-β/SMAD inhibitor) trigger transcytosis to cross the stromal barrier (173).

CAFs function as orchestrators of immunosuppression through secretion of immunosuppressive cytokines (TGF-β, IL-6, IL-10), chemokines that recruit regulatory T cells and MDSCs (CXCL12, CCL2), and metabolites that suppress T cell function (174, 175). All-trans retinoic acid (ATRA)-loaded nanoparticles represent the most extensively studied CAF/PSC quiescence-inducing platform. ATRA can restore activated PSCs to a quiescent state, reduce the secretion of CXCL12, thereby increasing the number of CD8^+^ T cells in the peritumoral area (176).

### 5.5 Biomarker-guided stratification and monitoring

Given the pronounced heterogeneity of PC, nano-immunotherapy is unlikely to be one-size-fits-all. The classification of the tumor microenvironment (TME) based on transcriptome analysis provides a framework for selecting patients to participate in prospective clinical trials of precision immunotherapy for PC (177, 178). **S**troma-high/immune-excluded tumors, characterized by high αSMA+ CAFs, FAP-α expression, collagen deposition and TILs (179), may preferentially benefit from stroma–immune co-modulation or localized/depot delivery to improve access. Myeloid-dominant tumors, identified by high CD68^+^CD163^+^ M2 macrophage infiltration, CD15^+^ARG1^+^ immunosuppressive granulocytes, and elevated CCL2/CCR2 axis activity, require TAM/MDSC-targeted reprogramming or recruitment blockade before applying checkpoint inhibitors (180). Whereas myeloid-dominant tumors may require TAM/MDSC-targeted reprogramming or recruitment blockade before applying checkpoint blockade. Tumors with weak innate priming—marked by low cGAS/STING expression, deficient type I interferon signatures, and minimal T-cell infiltration—may be better suited for localized delivery of innate agonists (e.g., STING/TLR agonists) combined with ICD-inducing therapies.

### 5.6 What has actually worked in PC nanomedicine and why (and what failed)

Despite thousands of preclinical studies and significant investments in the clinical translation of nanomedicines for PC, only two nanoformulations, namely nanoparticle albumin-bound paclitaxel (nab-paclitaxel, Abraxane^®^) and nanoliposomal irinotecan (Onivyde^®^), have been approved by the FDA and EMA for the treatment of pancreatic cancer (PC) (124,181). Nab–paclitaxel plus gemcitabine represents the most successful PC nanomedicine to date. In the landmark MPACT phase III trial (861 patients), the combination significantly improved median overall survival to 8.5 months versus 6.7 months with gemcitabine alone (HR 0.72, 95% CI 0.62–0.83, P<0.001), with 1–year survival rates of 35% versus 22% and 2–year survival of 9% versus 4% (182). Importantly, nab–paclitaxel demonstrated efficacy even in patients with poor performance status (ECOG 2), achieving 6–month survival rates of 63–69% with acceptable toxicity profiles (112). Nanoliposomal irinotecan (nal–IRI) achieved FDA approval in 2015 based on the NAPOLI–1 trial, which demonstrated that nal–IRI plus 5–fluorouracil/leucovorin significantly improved median overall survival to 6.1 months versus 4.2 months (HR 0.67, 95% CI 0.49–0.92, P = 0.012) in gemcitabine–refractory metastatic PDAC (183). The success of nab–paclitaxel and nanoliposomal irinotecan stems from addressing drug–specific pharmacokinetic limitations rather than relying solely on passive tumor targeting via the EPR effect (184). The success of nab–paclitaxel and liposomal irinotecan does not lie in attempting to create entirely new therapeutic mechanisms. Instead, based on passive tumor targeting relying on the EPR effect, they enhance drug delivery and efficacy by improving the biodistribution and pharmacokinetics of inherently active drugs with known clinical efficacy.

Unfortunately, there are still typical cases of failure in the field of nanomedicine for pancreatic cancer, which show significant potential in preclinical studies but fail in phase III clinical trials. PEGylated hyaluronidase (PEGPH20) in the HALO 109–301 trial failed to improve overall survival when combined with nab–paclitaxel/gemcitabine despite strong preclinical rationale for degrading hyaluronic acid–rich stroma. Similarly, evofosfamide (TH–302), a hypoxia–activated prodrug combined with gemcitabine in the MAESTRO trial, failed to demonstrate survival benefit (185). The fundamental obstacles to clinical translation are closely linked to the characteristics of PC, such as dense fibroproliferative stroma, vascular collapse, elevated interstitial fluid pressure, and insufficient perfusion. This unique adverse environment hinders the penetration of nanoparticles.

## 6 Factors affecting drug delivery

In the preceding section, we examined various types of targeting materials, including organic, inorganic, and biological nanoparticles, and their roles in PC DDSs. The properties of these carriers are critical for drug delivery. However, the therapeutic efficacy of targeted drugs depends not only on the carriers but also on the mechanisms by which the carrier targets the tumour.

### 6.1 Active and passive targeting

**Figure 4:**
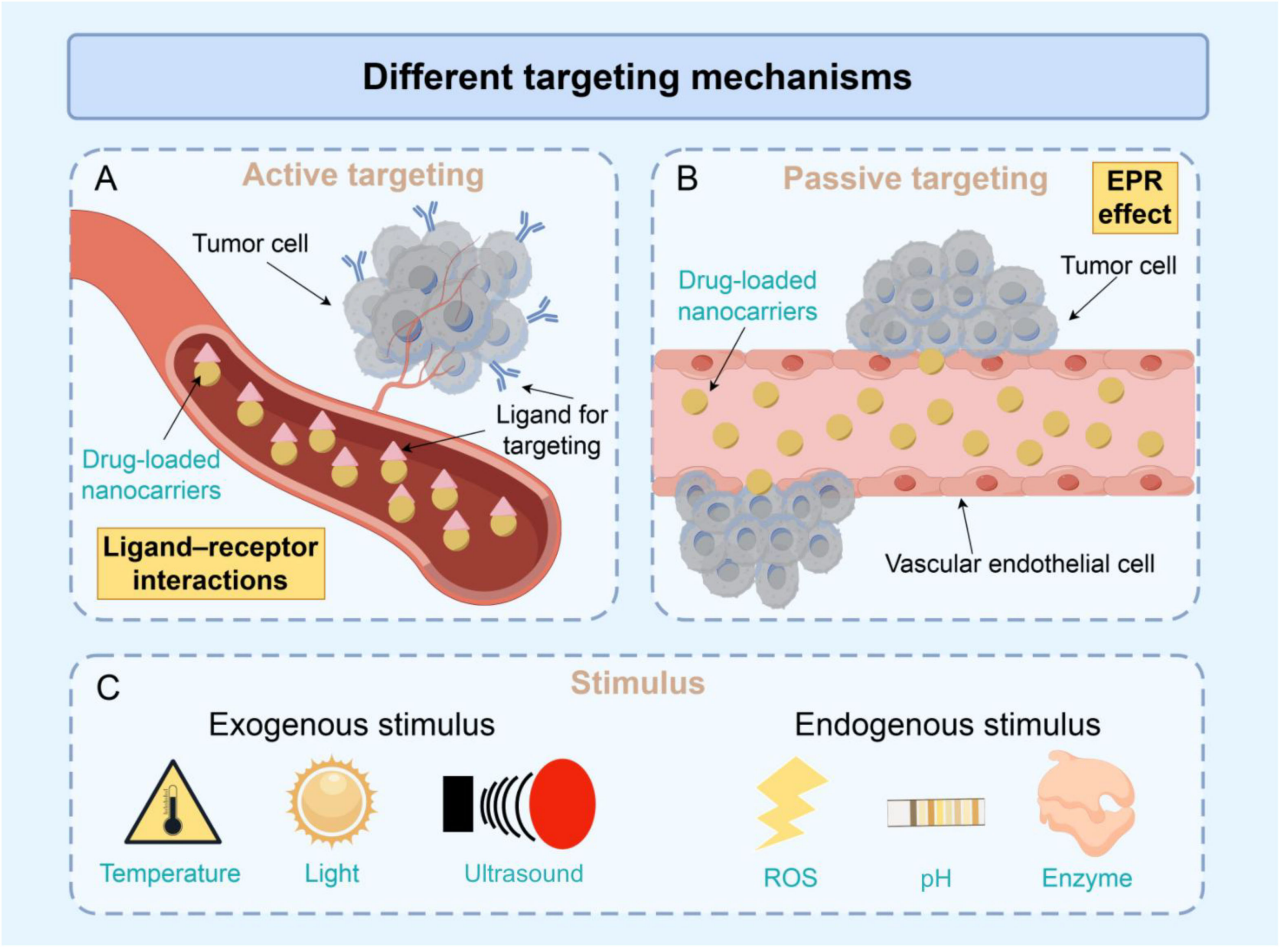
Factors affecting targeted drug delivery. Schematic illustration of major mechanisms governing nanocarrier delivery to pancreatic tumors: **(A)** active targeting driven by ligand–receptor interactions that promote cellular recognition and uptake, **(B)** passive targeting based on the enhanced permeability and retention (EPR) effect enabling extravasation of drug–loaded nanocarriers through tumor vasculature, and **(C)** stimuli–responsive delivery triggered by exogenous cues (temperature, light, ultrasound) or endogenous cues within the tumor microenvironment (reactive oxygen species (ROS), pH, and enzymes) to achieve on–demand drug release.

Active targeting (**Figure 4A**) involves specific molecular interactions between the surface molecules of the nanoparticles and receptors or proteins on the tumour, facilitating selective drug accumulation within tumour tissues and cells.

Molecular fragments can selectively bind to the surface of cancer cells and have many potential applications in imaging and therapy. Imaging agents that highlight tumour tissue and contrast it with healthy tissue aid in surgery and diagnosis, whereas active targeted therapies (e.g., antibody–drug conjugates (ADCs) and small–molecule ligands of cell surface receptors) facilitate tumour delivery and thus improve therapeutic efficacy. By understanding the biological characteristics of the cells to be killed, different types of ligands/molecules, such as peptides (186), antibodies (187, 188), proteins (189), polysaccharides (190), nucleic acids (191) and receptors (192), can be attached to the surface of the carrier or be applied to directly target to PC tissue (193), fibroblasts (194), KRAS mutant cells (195), tumour–associated macrophages (196), the tumour microenvironment (197), or nerves amongst PC cells (198) to increase the ratio of target to nontarget delivery. Drug–loaded nanocarriers can be designed by modification with targeting groups for preferential drug accumulation at the tumour site. New targeting agents and new targeting strategies will increase the delivery of chemotherapeutic drugs to specific sites. Targeting subcellular organelles such as mitochondria, nuclei, lysosomes, and the endoplasmic reticulum can provide maximum therapeutic effects while reducing the payload (199). Unfortunately, in active targeting, the highly selective interaction with cancer cells almost always occurs with a cell surface receptor, and the heterogeneity of tumours amongst a group of patients with cancer of the same organ increases the challenges of targeting (200).

In contrast, passive targeting (**Figure 4B**) leverages the unique physiological and pathological features of tumours along with the inherent properties of the nanoparticle system to promote efficient accumulation at the tumour site.

Impaired lymphatic drainage increases the accumulation of nanocarriers in the tumour area. This phenomenon is called the enhanced permeation and retention effect (EPR effect) (201, 202), which is the main force driving passive targeting. Nanomedicines are delivered to tumours mainly through the EPR effect after intravenous administration. To achieve maximum efficacy through passive targeting, nanoparticles must penetrate deep into the tumour and release their encapsulated drugs (199). Cholesterol–modified polymer CXCR4 antagonist (PCX) nanoparticles, which block cancer–stroma interactions, were designed to codeliver anti–miR–203, which inactivates matrix–producing pancreatic stellate cells (PSCs) and siKRAS^G12D^, which kills PC cells, were delivered to orthotopic syngeneic pancreatic tumours after intraperitoneal administration. Through their preferential localization to tumours and metastases, effective tumour penetration resulted in stromal destruction, delayed tumour growth, matrix depletion, reduced immunosuppression, inhibited metastasis, and prolonged survival (204). Unfortunately, owing to the heterogeneous permeability of tumours, some drugs do not diffuse effectively, which limits passive targeting. In addition, some tumours do not exhibit the EPR effect, which further hinders this process.

### 6.2 Exogenous and endogenous stimuli

DDSs can be specifically designed to respond to various stimuli, enabling targeted drug delivery and controlled release. Drug molecules are usually adsorbed/covalently bound to the surface of nanocarriers or encapsulated within them and released at the target site upon activation by an external energy field (temperature, light, or ultrasound) or a change in the local environment (such as pH, temperature, light, or ultrasound).

External energy fields (**Figure 4C**) can induce chemical reactions through artificial control and be applied to treat cancer to a certain extent (205) A study designed thermosensitive hydrogels encapsulating targeted nanoparticles for the local and sustained delivery of GEM and PTX to PC cells. After one week of sustained drug release, the growth of PANC–1 tumour spheroids was significantly reduced (206). In addition, this new photoresponsive nanoplatform targets the PC TME through tumour–specific mesokinin nanobodies (Nbs), which can effectively deliver semiconductor polymer nanoparticles (NPs) to the PC TME and generate a large amount of reactive oxygen species (ROS) locally under light excitation to achieve precise photoimmunotherapy.

The niche environment in which cancer cells reside is referred to as the cancer microenvironment and is closely related to the growth, invasion and metastasis of cancer cells. The cancer microenvironment has a variety of unique characteristics, such as varying pH values, expression levels of certain enzymes and redox environments (**Figure 4C**) (207). Some delivery systems are activated upon host–guest interactions in a certain state, antibody–antigen interactions, the overproduction of certain enzymes and changes in the microenvironment of the target tissue. GEM was loaded into 6PA–modified DGL (PDGL) nanoparticles to obtain PDGL–GEM, which was coprecipitated with the autophagy inhibitor chloroquine (CQ) and calcium phosphate to prepare PDGL–GEM@CAP/CQ. A change in the pH induced calcium phosphate dissolution, promoting the release of CQ from the nanobombs and the deep penetration of PDGL–GEM, which can inhibit PC proliferation and metastasis through an autophagy–dependent pathway (203). In terms of the enzyme response, a dynamic gelatine–hyaluronic acid hybrid hydrogel system was developed by integrating modular thiol–norbornane photopolymerization and enzyme (tyrosinase)–triggered on–demand matrix stiffening generating a photocrosslinked bioactive protein via thiol–norbornane gelation. Experiments revealed that in both HA–containing matrices and dynamically stiff microenvironments, this system inhibited PC cell growth (208). In addition, ROS–responsive nanoparticles have a wide range of applications. GEM–STING@Gel, a degradable reactive oxygen species–based hydrogel system, was designed to codeliver gemcitabine and the interferon gene stimulator DMXAA (5,6–dimethylxanthenone – 4 – acetic acid) to tumour sites and regulate the immunosuppressive TME by synergistically activating innate immunity and promoting the infiltration of cytotoxic T lymphocytes (209). In particular, trypsin and ROS coresponsive 11–mercaptoundecanoic acid–modified gold nanoclusters (MUA–Au NCs) were designed for targeted drug delivery to PC cells. These NCs target EGFR–overexpressing tumours and quickly deliver sufficient quantities of the drug to the tumour, subsequently increasing local methotrexate and ROS levels before being safely eliminated via the kidneys, making them more effective with fewer side effects than chemotherapy. Moreover, the treatment performance is improved in PC cells rich in trypsin (210).

Notably, TME–responsive nanoplatforms can be combined with exogenous light activation and imaging guidance to achieve spatiotemporally controlled multimodal therapy. For example, a hollow mesoporous MnO_2_–based, cell membrane–coated system enables TME–triggered carrier degradation with O_2_ generation and GSH depletion, while providing MRI/fluorescence guidance and single–laser PDT/PTT/CDT (211). Although demonstrated in a cervical cancer model, it exemplifies a generalizable design paradigm. In parallel, molecularly engineered carrier–free prodrug nanoassemblies represent an alternative strategy to improve stability and achieve controlled activation without relying on conventional liposomal or polymeric carriers. Feng et al. designed a library of doxorubicin prodrugs by linking DOX to fatty alcohols with different chain lengths via a tumor–responsive disulfide bond, enabling stable nanoassembly with tunable disassembly and release kinetics (212). Longer hydrophobic chains increased nanoassembly stability and prolonged circulation but slowed drug release, highlighting an explicit stability–activation trade–off. Although validated in a non–pancreatic tumor model, this study provides a representative example of how precise molecular design can balance circulation stability and rapid, triggerable activation at diseased sites.

## 7 Conclusion

Pancreatic cancer (PC) remains a devastating malignancy with rising incidence and persistently poor outcomes. The overall 5–year survival rate has improved modestly from approximately 5% to 13% over the past two decades, but this gain is driven almost entirely by improved outcomes in the small subset of patients diagnosed with early–stage, resectable disease, for the majority presenting with locally advanced or metastatic disease, survival remains largely unchanged (213, 214). Conventional therapies have provided only limited survival gains, largely because PC is protected by a uniquely hostile tumor microenvironment (TME) featuring dense desmoplastic stroma, profound hypoxia, poor perfusion, restricted CD8^+^ T–cell infiltration, and abundant immunosuppressive myeloid populations including tumor–associated macrophages (TAMs), myeloid–derived suppressor cells (MDSCs), and regulatory T cells (122, 125, 133, 215). Collectively, these features create an immunologically “cold” tumor that is largely unresponsive to checkpoint inhibitor immunotherapy and resistant to cytotoxic chemotherapy (125, 216).

Accordingly, exploiting TME–associated cues and cellular interactions has become a central direction for next–generation therapy. Targeted drug delivery systems (DDSs)—spanning organic, inorganic, and biological platforms including liposomes/lipid nanoparticles, polymeric systems, carrier–free drug self–assembly nanoparticles, hybrid inorganic–organic nanomaterials, and biomimetic carriers such as exosomes and protein nanocages—can increase local drug exposure while reducing systemic toxicity through ligand–receptor targeting, microenvironment–adaptive transport, and controlled release triggered by internal stimuli (pH, enzymes, reactive oxygen species, hypoxia) or external stimuli (ultrasound, light, magnetic fields). Beyond treatment, these technologies also provide opportunities for theranostic integration and response monitoring.

Importantly, the future of PC nanomedicine should not be framed solely around improving cytotoxic delivery. DDSs are designed to remodel the immunosuppressive TME by reprogramming tumor–associated macrophages, limiting MDSC recruitment and function, activating innate immune sensing pathways such as cGAS–STING, and addressing the stroma not only as a physical barrier but also as an immunological shield that enforces immune exclusion. Strategies that couple stromal modulation with enhanced T–cell infiltration and immune activation are especially promising for converting “cold” PC into immunotherapy–responsive disease, particularly in rational combinations with checkpoint blockade and immunogenic cell death–inducing therapies. In parallel, localized or depot delivery approaches may offer practical advantages for deep–seated pancreatic tumors by improving intratumoral exposure and limiting systemic adverse events.

Given the marked heterogeneity of PC, progress toward meaningful clinical benefit will also require a precision–medicine framework. Biomarker–guided stratification and monitoring using transcriptomic and TME–feature signatures can help match patients to the most appropriate DDS modality and combination regimen, and enable longitudinal assessment of immune remodeling. Meanwhile, advances in bioinformatics and artificial intelligence/machine learning are expected to accelerate carrier design, optimize multi–agent co–delivery, and identify actionable biomarkers that predict delivery efficiency and therapeutic response (217). In addition, beyond scientific performance, reimbursement and pricing increasingly shape real–world adoption of complex DDSs. Biomarker–guided enrichment of responders may improve not only therapeutic outcomes but also the cost–effectiveness of advanced DDS regimens, thereby facilitating reimbursement and real–world adoption (218).

From a translational standpoint, many DDS programs fail due to CMC and regulatory issues rather than lack of efficacy, including poor batch reproducibility, unstable drug loading/release, scale–up/sterilization incompatibility, and immunotoxicity (219). A minimal, fit–for–purpose IND–enabling package typically includes defined critical quality attributes (size/PDI, drug content/loading, impurities/endotoxin), stability and *in vitro* release in relevant matrices, reproducible manufacturing with justified specifications, PK/biodistribution, and preliminary safety/toxicology (including immunotoxicity) (220, 221). We highlight these as practical checkpoints to improve translatability.

In summary, PC therapy is moving toward more sophisticated DDSs that integrate targeted delivery, controlled release, immunomodulation, and patient–specific selection. Despite strong preclinical momentum, major barriers remain, including variable delivery efficiency in stroma–rich tumors, safety considerations for potent immune agonists, and challenges in reproducible manufacturing, scalability, and regulatory translation (222, 223). Continued innovation, standardized evaluation across clinically relevant models, and well–designed clinical trials will be essential to validate safety and efficacy and to realize the full potential of next–generation, precision DDS strategies for pancreatic cancer (224).
